# Translation and validation of the Arab version of the Late-Life Function and Disability Instrument: a cross sectional study

**DOI:** 10.1186/s12877-015-0046-8

**Published:** 2015-04-24

**Authors:** Michal Elboim-Gabyzon, Maayan Agmon, Faisal Azaiza, Yocheved Laufer

**Affiliations:** Physical Therapy Department, Faculty of Social Welfare and Health Sciences, University of Haifa, Haifa, Israel; Department of Nursing, Faculty of Social Welfare and Health Sciences, University of Haifa, Haifa, Israel; School of Social Work, Faculty of Social Welfare and Health Sciences, University of Haifa, Haifa, Israel

**Keywords:** Reliability, Validity, Arabic, Function, Disability, Elderly

## Abstract

**Background:**

The Late-Life Function and Disability Instrument (LLFDI) provides a comprehensive, reliable, and valid assessment of physical function and disability in community-dwelling adults. There does not appear to be a validated, comprehensive instrument for assessing function and disability in Arabic. The objective of the present study was to translate and culturally adapt the LLFDI to Arabic, and to determine its test-retest reliability and validity.

**Methods:**

The LLFDI was translated to Arabic through a forward and backward translation process, and approved by a bilingual committee of experts. Sixty-one (26 male and 35 female) Arabic speaking, healthy, older adults, ages 65–88, living in northern Israel participated in the study. To determine test-retest reliability, the questionnaire was administered twice to 41 subjects with a 6 to 8day interval. Construct validity was examined by correlating the LLFDI responses with the 10-item physical function (PF-10) subscales of the General Health Survey (SF-36), with the physical component of SF-36 (SF-36 PCS), and with two performance measures, the Berg Balance Scale (BBS) and Time Up and Go (TUG) test. Additionally, gender and fall related differences in the LLFDI were also examined.

**Results:**

Internal consistency (Cronbach’s alpha) was good to excellent (0.77 to 0.97). Test-retest agreement was good to very good (function component: 0.86–0.93, disability component: 0.77–0.93). Correlation with the SF-36 PCS and PF-10 was moderate to strong for both LLFDI components (function, r = 0.53–0.65 and r = 0.57–0.63, and LLFDI disability, r = 0.57–0.76 and 0.53–0.73, respectively). Significant, moderate-to-strong correlations between the LLFDI and BBS (r = 0.73–0.87) and a significant, moderate, negative correlation between LLFDI and TUG test (r = −0.59– -0.68) were noted. The standard error of measure was 6–12%, and the smallest real difference was 18–33%. Discriminative validity for both gender and fall status were also demonstrated.

**Conclusions:**

The Arabic version of the LLFDI is a highly reliable and valid instrument for assessing function and disability in community dwelling, Arab older adults. The translated instrument has a discriminative ability between genders and between fallers and non-fallers. The translated instrument may be used in clinical settings and for research purposes.

## Background

Age related physiological changes and greater susceptibility to disease and chronic conditions often affect the physical, social and psychological wellbeing of older adults [1]. With the rapid increase in the proportion of older adults within the general population, maintaining functional ability and independent living of the aging population are important health objectives worldwide [2,3]. Reliable and valid assessment of functional performance and disability in later life is essential for estimating the impact of aging and disease on the well-being of older adults, which is necessary for planning rehabilitation and support services and for monitoring the effectiveness of intervention programs.

Functional performance reflects an individual’s capacity to perform a variety of tasks relevant to community living, such as walking, ascending and descending stairs, reaching, and handling everyday objects. Disability refers to a person’s ability to carry out socially defined life tasks expected of an individual within a typical socio-cultural and physical environment [4]. Physical performance-based tests, such as gait velocity assessment, are frequently used to assess specific functional limitations due to their high reliability and ability to predict disability and mortality [5,6]. However, self-report measures are often preferred owing to their low cost and practicality [7]. Furthermore, self-reports also reflect an individual’s subjective perceptions regarding performance capabilities and achievements within one’s personal socio-cultural context [8].

Although a variety of self-report instruments are available for clinical and research purposes, most do not offer a comprehensive assessment of function and disability and are criticized for lacking a theoretical framework and for poor psychometric properties [9-11].The Late-Life Function and Disability Instrument (LLFDI) was developed in 2002 to address some of these limitations [12,13]. Based on Nagi’s Disablement Model [8] and the World Health Organization’s International Classification of Functioning, Disability and Health [14], it is comprised of two components relating to physical function and disability. The LLFDI has been used in numerous studies since its development. A recent systematic review of the psychometric properties of the LLFDU identified 71 studies including over 17,000 community dwelling older adults that had utilized this instrument. High test-retest reliability was found for the functional component, with the results for the disability component more variable. The review indicated the existence of evidence supporting the construct validity and sensitivity to change of the LLFDI [7].

It is generally accepted that cross-cultural adaptation of validated assessment instruments is not only more efficient than developing new self-report measures in each language, but also allows researchers to pool data from trials conducted with populations whose native tongue is different [15]. While the LLFDI has been translated from English and validated in several languages, including Spanish [16], Swedish [17], and Hebrew [18], there appears to be no validated comprehensive assessment of function and disability instruments in Arabic. Arabic is one of the five most widely spoken languages in the world, and its popularity is expected to increase in coming years, and not only within the boundaries of Arab countries [19]. Thus, the objective of the present study was to translate and culturally adapt the LLFDI to Arabic, and to determine its test-retest reliability and construct validity among Arab-speaking, community dwelling, older adults.

## Methods

### The Late-Life Function & Disability Instrument (LLFDI)

The LLFDI was designed to achieve a comprehensive assessment of physical function and disability in community-dwelling, older adults [7,12,13]. The function component of the LLFDI assesses self-reported difficulties in performing 32 physical activities and is comprised of three domains: (1) upper extremity (items that reflect activities of the hands and arms), (2) basic lower extremity (items that reflect activities primarily involving standing, stooping, and fundamental walking activities), and (3) advanced lower extremity (items that reflect activities that involve a high level of physical ability and endurance).Questions are phrased, “How much difficulty do you have doing a particular activity without the help of someone else and without the use of assistive devices?” Each of the questions is graded on a five-point Likert scale ranging between 1–5 with the response options of “cannot do,” “quite a lot,” “some,” “a little,” “none”.

The disability component includes limitations and frequency dimensions. The limitations dimension assesses the individual’s self-perceived limitations in taking part in 16 major life activities. This part includes two domains: (1) instrumental role (including items that reflect limitation in activities at home and in the community), and (2) management role (including items that reflect limitation in organization or management of social tasks that involve minimal mobility or physical activity). Limitation questions are phrased, “To what extent do you feel limited in doing a particular task?” Each of the questions is graded on a five-point Likert scale ranging between 1–5 with the response options of “completely”, “a lot,” “somewhat,” “a little,” and “not at all,”.

The frequency dimension of the disability component addresses the individual’s self-perception of the frequency of performing these 16 tasks regularly. This part includes two domains: (1) social role –various social and community tasks, and (2) personal role –various personal tasks. Frequency questions are phrased, “How often do you do a particular task?” Each of the questions is graded on a five-point Likert scale ranging between 1–5 with the response options of “never,” “almost never,” “once in a while,” “often,” and “very often”.

The overall raw scores and the raw scores for each component, dimension and domain were transformed to scaled scores (0–100) using the score tables provided by the developers (Royal Center for Enhancement of Late-Life Function, Sargent College of Health and Rehabilitation Sciences, Boston University), which are based on a Rasch analysis model [13].

### Translation and cross-cultural adaptation

The LLFDI instrument was translated by the forward and backward translation procedure following recommended guidelines [20]. Forward translation into Arabic was carried out independently by one bilingual professional translator (native Arabic-speaking) and one bilingual physical therapist, familiar with the terminology. The translators were requested to use standardized literary Arabic. The translations emphasized conceptual and cultural meanings rather than literal translations. Thus, all references to miles were converted to kilometers, and several of the examples were modified in accordance with local culture. For example, in order to describe reaching overhead we used the example of reaching to a high shelve instead of giving the original example of pulling the string of a lamp (which is not a common local feature). Another example relates to taking care of local errands, whereas going to the dry cleaner or library was changed to going to the mail box and the local health center. A group discussion of an expert panel including five bilingual physical therapists with clinical expertise in the area of elderly care, led by two of the researchers (MEG and MA), was used to compare the versions. Inadequate expressions and concepts were identified and resolved. The backward translation into English was conducted by two independent bilingual translators who did not participate in the previous stages. A consensus regarding the accuracy of the version translated back into English was reached by two of the researchers (MEG and YL). The final Arabic version was pre-tested with ten elderly persons who did not participate in the test-retest study. They were asked whether the questions were clear and relevant to their life situation. The group reported that all items were well understood.

### Participants and psychometric properties of the scale

The study was approved by the Institutional Review Board of the Faculty of Social Welfare and Health Sciences at the University of Haifa. The subjects were recruited by snow ball sampling from three geriatric community centers, with one located in a city and two in Arab villages. All patients were informed about the nature of the study and gave written informed consent before participation.

To assess the validity, the final Arabic version of the LLFDI was administered to a convenience sample of 61 elderly people within the Arab population of northern Israel. The inclusion criteria were age above 65 years, ability to understand simple commands, able to walk independently without using a walking aid, no major orthopedic or other medical disorder that might affect independent living, and no serious uncorrected visual or hearing impairments.

Test-retest reliability was determined by administering the questionnaire twice to 41 of the subjects with a 6 to 8day interval between tests. This interval was chosen both to avoid variations in clinical status and to avoid the patient remembering previous answers. In addition, the subjects were asked to report whether anything had happened during this interval that could influence their response to the questionnaire.

After completing the LLFDI, the subjects were requested to complete the Short Form General Health Survey (SF-36) and two performance-based clinical measures of balance and gait: the Berg Balance Scale (BBS) and the Time Up and Go test (TUG).

The SF-36 Physical Component Score (SF-36 PCS) is one of the two main subscales of the SF-36 and represents health status in terms of physical function, role-physical, pain and general health [21]. It is reported to have good internal consistency and test-retest reliability [21,22]. The 10-item Physical Function (PF-10) scale which is part of the SF-36, encompasses three main attributes of physical function: (1) self-care (2) mobility and (3) body movement, such as bending and lifting [23]. The SF-36 PCS and PF-10 have been proven valid and reliable [23,24]. Previous studies used these tools to validate the original English version and the translated versions of the LLFDI [7].

The Berg Balance Scale (BBS) is a 14-item measure designed to assess static balance and fall risk in adult populations [25]. The subject is asked to perform static and dynamic activities of varying difficulty. Item-level scores range from 0–4 with higher scores indicating better performance [25,26]. A score less than 45 indicate that individuals may be at a risk of falling [27].

The Time Up and Go test (TUG) is a very common valid, reliable tool to assess mobility, balance, and walking ability in community dwelling adults [26]. The test measures the time that is required for a subject to rise from a chair, walk 3 meters at a safe, comfortable pace, turn around, walk back to the chair and sit down [28]. It was reported that a TUG score less than 20 seconds indicates independence in basic transfers in community dwelling elderly people, while scores higher than 30 in this population indicate dependence during transfers, difficulties in entering and exiting a shower or tub, and in outdoor ambulation [28]. A score higher than 13.5 is the cut off that indicates risk of falls in community dwelling adults [27].

Construct validity was examined by correlating the overall scores and each LLFDI subscale with the physical component of the SF-36 (SF-36-PCS) and the 10 item Physical Functioning Subscale (PF-10) of the SF-36, as well as with the two performance measures.

Discriminate validity was assessed by comparing the LLFDI scores between fallers and non-fallers. The fall status was ascertained by self-report of number of falls in the past year. Individuals reporting one or more falls within the previous year were considered as fallers.

### Statistical analysis

Basic demographic and clinical characteristics, and results of the performance tests (TUG, BBS) and the SF-36 components were reported as mean and standard deviation (SD), or counts and percentages, as appropriate. The LLFDI results were characterized by mean and SD. Internal consistency was determined with Cronbach’s alpha with α values ≥ 0.9 considered excellent, 0.7 ≤ α < 0.9 considered good, 0.6 ≤ α < 0.7 acceptable, 0.5 ≤ α < 0.6 poor, and values of α < 0.5 unacceptable [29]. Test-retest reliability was determined with ICC_2,1 _and classified according to Bland and Altman [30], that is ≤0.20 poor, 0.21-0.40 fair, 0.41-0.60 moderate, 0.61-0.80 good, 0.81-1.00 very good. Absolute reliability was analyzed both by the standard error of measure (SEM) and by the smallest real difference (SRD). SEM was determined as the standard deviation of the first test session scores X square root (1 – ICC_2,1_). SRD was determined as 1.96 × SEM X square root (2), that is, 2.77 × SEM. Also calculated were percentage SEM (SEM%) and percentage SRD (SRD%). SEM denotes the smallest change that indicates a real difference for a group of subjects, while SRD represents the smallest change that indicates a real improvement for a single subject [31,32].

Concurrent validity of the LLFDI components was evaluated using Pearson correlation with the SF-36 PCS, PF-10, BBS, and TUG. Consistent with the criteria reported by Portney and Watkins [33], Pearson correlation (r) coefficients above 0.75 represent a strong correlation, and the values ranging between 0.50 to 0.75 suggest a moderate correlation.

Gender differences and differences between fallers and non-fallers in the LLFDI were examined by t-test. Statistical analyses were performed using JMP (SAS Institute, Cary, NC) and Excel (Microsoft Corp., Redmond, WA).

## Results

Sixty-one (26 male and 35 female) volunteers participated in the study. The sample included 34 non-fallers (55.7%) and 27 fallers (44.3%) who had fallen 1 to 6 times in the year preceding data collection. Table 1 describes the demographic characteristics of the subjects. The results of the SF-36 PCS, the PF-10 and the performance based clinical measures of balance and gait are also presented in Table 1. Results (mean ± standard deviation) of the LLFDI at both assessment periods are presented in Table 2. Table 3 presents the internal consistency and reliability analysis of the function and disability components as well as of all the dimensions and domains.

**Table 1:** **Demographic and clinical characteristics of the study sample (n = 61)**

**Characteristic**	**Mean ± SD or Number (%)**
Age, years	74.1 ± 6.2
Gender: Male, Female	26 (42.6%), 35 (57.4%)
Religion: Christian, Moslem, Druze	26 (42.6%),17 (27.9%), 18 (29.51)
Number of children	5.9 ± 3.1
Family status: Married, Widowed, Not married	41 (67.21%), 17 (27.87%), 3 (4.92%)
Height, cm	160.9 ± 10.3
Body mass index, kg/cm^2^	29.5 ± 5.6
Medical history: Comorbidities, Medications prescribed, Previous surgeries	1.6 ± 1.2, 3.1 ± 2.6, 1.3 ± 1.3
Physical component score (SF-36 PCS), 0-400	242.5 ± 107.2
Physical functioning subscale (PF-10), 0-100	61.8 ± 30.2
Time Up and Go test, sec	15.2 ± 7.3
Berg Balance Scale, 0-56	43.7 ± 12.4

**Table 2:** **The results of Late-Life Function & Disability Instrument over time (mean ± SD)**

**Test results**	**Test 1 (n = 61)**	**Test 2 (n = 41)**
**Function component**		
**Function total**	61.8 ± 3.0	65.6 ± 2.9
Upper extremity	81.2 ± 11.0	86.8 ± 11.5
Basic lower extremity	73.7 ± 8.0	79.5 ± 9.2
Advanced lower extremity	51.9 ± 5.0	56.9 ± 4.4
**Disability component**		
**Frequency total**	45.5 ± 3.0	50.2 ± 3.3
Social role	41.4 ± 4.5	46.8 ± 4.7
Personal role	48.1 ± 5.5	53.4 ± 6.3
**Limitation total**	70.5 ± 7.3	80.6 ± 10.1
Instrumental role	69.3 ± 8.3	79.7 ± 10.8
Management role	80.0 ± 11.1	89.2 ± 13.4

**Table 3:** **Results of the reliability analyses and of internal consistency**

**PARAMETER**	**MEAN DIFFERENCE**	**LOWER 95% CI MD**	**UPPER 95% CI MD**	**LOWER 95% CI ICC(2,1)**	**UPPER 95% ICC(2,1)**	**ICC(2,1)**	**SEM**	**SEM%**	**SRD**	**SRD%**	**Cronbach’s α**
**Function component**											
**Function total**	−1.2	−3.1	0.6	0.84	0.95	0.91	4.4	67	112.2	118	0.98
Upper ext.	0.2	−2.7	3.0	0.75	0.92	0.86	6.7	78	118.5	221	0.93
Basic lower ext.	−1.0	−3.3	1.4	0.85	0.95	0.92	5.1	6	14.2	118	0.97
Adv. lower ext.	−1.5	−3.6	0.6	0.88	0.96	0.93	45.0	8	113.7	223	0.94
**Disability component**											
**Frequency total**	1.2	−1.0	3.4	0.72	0.91	0.84	4.6	19	112.8	226	0.9
Social role	1.6	−0.9	4.1	0.75	0.92	0.86	5.4	12	114.9	333	0.85
Personal role	1.0	−2.0	4.0	0.61	0.87	0.77	6.3	112	117.5	333	0.77
**Limitation total**	2.7	0.1	5.3	0.86	0.96	0.93	56.1	78	16.9	222	0.97
Instrumental role	2.4	−0.4	5.1	0.87	0.96	0.93	6.3	8	17.4	223	0.97
Management role	3.3	0.6	6.0	0.80	0.95	0.90	67.0	78	119.4	1123	0.81

CI-confidence interval, MD-Mean Difference, ICC2.1- intra-class correlation coefficient, SEM -standard error of measure, SEM%-coefficient of variance, SRD-smallest real difference, SRD%- smallest real difference %, Ext. - Extremity, Adv.- Advanced.

### Internal consistency

#### Function component

The internal consistency of the function component of the LLFDI was excellent, with Cronbach’s α values ranging from 0.93 to 0.97.

#### Disability component

The disability component demonstrated good to excellent internal consistency, with Cronbach α values ranging from 0.77 to 0.97 which is somewhat lower than the values of the function component. The lowest internal consistency was found for the personal and management roles (Cronbach’s α 0.77 and 0.81 respectively).

### Reliability

#### Function component

Very good test-retest reliability was found for the disability component with the ICC_2,1_ ranging from 0.80 to 0.93. The personal role was the only exception, with an ICC_2,1_ in the good range (0.77). Absolute reliability showed that the measurement error at the group level (SEM) was 4. 6–7 (8–12%). The measurement error at the individual level (SRD) was 12.8–19.4 (22–33%).

#### Disability component

Very good test-retest reliability was found for the disability component with the ICC_2,1_ ranging from 0.80 to 0.93. The personal role was the only exception, with an ICC_2,1_ in the good range (0.77). Absolute reliability showed that the measurement error at the group level (SEM) was 4. 6 –7 (8–12%). The measurement error at the individual level (SRD) was 12.8–19.4 (22–33%).

### Concurrent Validity

#### Function component

The function component, and its domains demonstrated significant moderate-to-strong correlations with BBS (r = 0.74 to 0.87, p < 0.0001) and a moderate, significant negative correlation with the TUG test (r = −0.62 to −0.68, p < 0.0001). The LLFDI function component demonstrated moderate correlation with the SF-36 PCS and PF-10 (r =0.53 to 0.65, p < 0.0001). In general, the strongest evidence for construct validity between the LLFDI and the BBS and the TUG was noted for the basic lower extremity domain (r = 0.87, −0.72, respectively). See Table 4.

**Table 4:** **Results of the Pearson analysis (ICC)**

**Component**	**BBS**	**TUG**	**SF-36 PCS**	**PF-10**
**Function component**				
**Function total**	0.80	−0.68	0.65	0.63
Upper extremity	0.77	−0.62	0.53	0.57
Basic lower extremity	0.87	−0.72	0.63	0.60
Advanced lower extremity	0.74	−0.66	0.65	0.61
**Disability component**				
**Frequency total**	0.75	−0.63	0.58	0.58
Social role	0.74	−0.61	0.57	0.60
Personal role	0.73	−0.59	0.56	0.53
**Limitation total**	0.81	−0.61	0.74	0.70
Instrumental role	0.83	−0.61	0.76	0.73
Management role	0.76	−0.64	0.62	0.56

BBS-Berg Balance Scale, TUG- Time Up and Go test, SF-36 PCS -SF-36 Physical Component Score, PF-10-10-item Physical Functioning subscale.

The results were significant p < 0.001.

#### Disability component

The disability component demonstrated significant moderate-to-strong correlations with the BBS (r = 0.73 to 0.83, p < 0.0001) and a moderate, significant negative correlation with the TUG test (r = −0.59 to −0.64, p < 0.0001). A moderate-to-strong significant correlation was demonstrated with the SF-36 PCS and PF-10 (r = 0.53-0.76, p < 0.001). See Table 4.

### Discriminative validity

Gender related differences were observed in the two components, the dimensions and the domains of the LLFDI, with the male subjects reporting higher function, and lower level of disability compared to females. The only exception was in the personal role domain, in which the men and woman showed no difference in the frequency of participating in various personal tasks (see Table 5). Significant differences between fallers and non-fallers were demonstrated for the function and disability components with the non-fallers demonstrating higher function and lower level of disability. see Table 5.

**Table 5:** **Comparison between the results of Late-Life Function & Disability Instrument in terms of gender and fall status**

**Components**	**Gender**			**Falling status**		
	**Male (n = 26)**	**Female (n = 35)**	**p value**	**Fallers (n = 27)**	**No fallers (n = 34)**	**p values**
**Function component**						
Function total	68.5 ± 14.9	56.8 ± 15.5	0.005	56.2 ± 14.5	66.5 ± 16.3	0.01
Upper extremity	90.4 ± 18.7	74.2 ± 21.5	0.003	75.1 ± 22.2	86.2 ± 20.3	0.049
Basic LE	81.9 ± 17.2	67.4 ± 21.2	0.006	65.4 ± 19.1	80.4 ± 19.8	0.005
Advanced LE	60.4 ± 18.4	45.4 ± 21.3	0.006	44.0 ± 21.1	58.4 ± 19.5	0.008
**Disability component**						
Frequency total	49.4 ± 13.8	42.6 ± 8.8	0.02	41.7 ± 8.2	48.6 ± 13.0	0.02
Social role	47.3 ± 15.5	37.0 ± 11.5	0.004	35.8 ± 11.6	45.8 ± 14.6	0.005
Personal role	50.5 ± 15.3	46.2 ± 11.8	0.21	44.9 ± 10.8	50.5 ± 14.9	0.1
Limitation total	77.2 ± 20.9	65.5 ± 23.5	0.049	61.7 ± 22.1	77.4 ± 21.6	0.007
Instrumental role	76.8 ± 22.8	63.7 ± 26.5	0.048	59.1 ± 25.1	77.3 ± 23.4	0.005
Management role	87.3 ± 18.6	74.6 ± 22.6	0.02	72.8 ± 25.5	85.7 ± 18.7	0.02

LE – lower extremity.

## Discussion

This study examined the psychometric properties of the LLFDI translated into Arabic as assessed with Arabic speaking, community dwelling, older adults, ages 65–88 years, living in northern Israel. The internal consistency of the Arab version of the LLFDI function component and its domains were excellent and comparable to the results reported by Haley et al. for the original English version [12], and by Roaldesen et al. [17] for the Swedish version. The internal consistency of the total score of the two dimensions of the disability component were excellent as well, and were consistent with the results by Hand et al. [24] who tested middle aged (45–65 years), community-dwelling adults with chronic health conditions, and with the results of Roaldsen et al. [17] who examined older community-dwelling adults (aged 68–88 years). However, the internal consistency of both dimensions of the disability component of the version translated into Arabic were somewhat higher than those the original English version studied by Jette et al. [13]. It should be noted that to the best of our knowledge, this is the first study to examine the internal consistency within each domain in a version other than the original English questionnaire.

The test-retest reliability of the two components of the Arabic version of the LLFDI was moderate-to-high. The high test-retest reliability (ICC_2,1_) values of the function component were comparable with the results of the English and the Swedish versions [12,17]. The test-retest reliability values of the LLFDI disability component were slightly higher than those found for the original English by Jette et al. [13] and Haley et al. [12], but similar to the results of the Swedish version of the LLFDI [17]. Similar to previous studies, the test-retest reliability values of the function component were higher compared to the test-retest reliability values of the disability component [7,13,17,34].

To the best of our knowledge, only one other study examined the absolute reliability of the LLFDI [17]. However, scores of the domains of the disability component (social, personal, instrumental and management roles) have not been previously reported. Comparing the current results to this study indicates similar absolute reliability in the function component, while the scores of the frequency and limitation dimensions of the disability component were slightly higher in the present study. The relatively low SEM% and SRD% suggest reasonably high inter-test precision, a pre-requisite for sensitivity to individual or group changes in longitudinal studies.

Correlation analysis was used to determine construct validity between the translated LLFDI and both performance based measures and two aspects of the SF-36 self-report questionnaire. Generally, both the function component and the limitation dimension demonstrated values of ICC_2,1_ in the good range with all outcome measures tested. Somewhat lower correlations were found for the frequency dimension. Higher correlations were determined with the BBS in comparison to the correlation with the other outcome measures. These correlation values were higher than those demonstrated in previous studies. For example, Melzer et al. [35] reported a significant correlation between the LLFDI and BBS (r = 0.48) and the TUG (r = −0.52) only for the function component. However, it should be noted, that our subjects had overall poorer balance capabilities as demonstrated by lower scores on both the BBS and the TUG, with the mean score of the BBS indicating they were at risk for falling. The correlation between the function and disability component of the LLFDI and performance based measures was higher than the correlation with the self-report measures, with only one exception (the correlation between the limitation dimension and the TUG test). This may be related to the fact that the underlying constructs being measured in the LLFDI and the performance based measures are more closely related. Similar results were found in a systematic review done by Beauchamp et al. [7] on the psychometric properties of the LLFDI.

All the test-retest reliability measures, as well as the validity measures were lower for the personal role domain within the frequency dimension. These lower values may be due to the fact that the personal role domain includes only seven items, leading to poorer psychometric properties [34].

Consistent with previous studies, discriminative validity was demonstrated, as both the function and disability components discriminated between fallers and non-fallers, with the non-fallers demonstrating higher function and low level of disability [7,18].

To the best of our knowledge, normative values are not available for the LLFDI in community dwelling older adults [34] and previous studies have not examined gender related differences for the LLFDI. The current study showed significant differences between genders in both the function and disability components and for all domains except for the personal role domain. Generally, higher scores in both the function and disability components were demonstrated by the elderly man compared to the woman. Other assessment instruments have demonstrated similar gender related differences in function among the elderly, with women consistently reporting greater functional difficulties than men [36]. Women were more likely to report limitations, need for assistance, and greater degree of disability [37,38]. These gender difference are not well understood, but it has been suggested that they may be related to differences in disability-related health conditions [38], or/and to differences in self-perception stemming from social and cultural factors [36].

The personal role domain which includes items such as performing errands, meal preparation, personal care needs, and taking care of household business, is probably more culturally and socially dependent than most of the domains covered by the LLFDI. In the traditional Arab culture there is a distinguishable role division between genders [39]. Arab women, especially the older generation, are generally expected to be primarily responsible for household chores. The husband usually fulfills the dominant instrumental roles as primary provider and is responsible for most outdoor chores [40]. Given this, one would have expected significant differences between the female and male respondents for personal role domain, which was not demonstrated in this study. However, as the Arab culture is characterized by a multi-generational household, younger female family members often assist the elderly women with household work [41]. This might have masked the expected differences between genders for this domain. Further studies are necessary to illuminate these findings.

Some limitations of the study must be considered. The translated version used standard literary Arabic. Given the many dialects of the Arabic language, some terms may differ between one Arab country to another and even from one region to another in the same country. Furthermore, some of the participants required help reading the questionnaire and were dependent on help provided by the person administering the questionnaire which may have introduced some bias. Finally, the studied participants were heterogeneous in terms of health condition, age and body mass, which probably affected results.

## Conclusions

The current study demonstrated that the Arab version of the LLFDI has good internal consistency, moderate to high test retest reliability and good construct and discriminative validity. These results indicate that the translated version of the LLFDI can be used as a tool to screen for disability, develop health policies, and design appropriate intervention programs for Arab speaking, community living adults.
